# Design and mechanical damage behavior of radially gradient-controllable Ti6Al4V bionic porous bone scaffolds

**DOI:** 10.3389/fbioe.2026.1928634

**Published:** 2026-09-15

**Authors:** Yuxi Liu, Shuge Li, Aihua Li, Yinhui Zhong, Bing Du, Tianshun Liu

**Affiliations:** 1 School of Smart Health, Chongqing Polytechnic University of Electronic Technology, Chongqing, China; 2 Department of Gastroenterology, Chongqing University Cancer Hospital, School of Medicine, Chongqing University, Chongqing, China; 3 School of Vehicle Engineering, Chongqing Industry Polytechnic University, Chongqing, China; 4 Chongqing Key Laboratory of Nano-Micro Composite Materials and Devices, School of Metallurgy and Materials Engineering, Chongqing University of Science and Technology, Chongqing, China

**Keywords:** biomimetic design, mechanical behavior, porous scaffold, SLM 3D printing, tree-like fractal

## Abstract

Inspired by the striking structural resemblance between trabecular bone microarchitecture and the dendritic branching morphology, we propose a novel biomimetic bone scaffold design strategy that replicates tree-derived fractal architecture. Four distinct fractal-structured porous scaffolds were fabricated via Selective Laser MeltingSLM 3D printing technology using Ti6Al4V alloy. The porosity distribution, damage mode, and mechanical properties of these scaffolds were systematically investigated. Through systematic modulation of fractal dimension and branching parameters, biomimetic bone scaffolds with customizable pore architecture and spatial distribution characteristics can be precisely fabricated. The porosity of the biomimetic scaffold gradually increases from the outer to the inner region, mimicking the porosity distribution of native bone. The biomimetic scaffolds with third-order fractal exhibit excellent mechanical properties of high compressive damage resistance and low elastic modulus. The compressive damage evolution pattern of biomimetic porous scaffolds is highly consistent with that of natural bone. The fracture propagation path presents an approximately 45° angle relative to the scaffold axis, which endows the scaffold with enhanced axial load-bearing capacity. The bionic porous scaffold designed in this study achieves consistent porosity distribution and analogous mechanical responses with natural bone.

## 1 Introduction

The clinical healing of large segmental bone defects remains a significant clinical challenge in orthopedic practice (Tian et al., 2025), profoundly affecting patients’ health and quality of life (Acar et al., 2024; Abd-Elaziem et al., 2024). Most large bone defects arise from congenital abnormalities, traumatic injuries, or bone tumors (Zhou et al., 2019; Pobloth et al., 2018). A bone defect is defined as “large segmental” if it exceeds 50% of the bone diameter or 2 cm in length (Shen et al., 2022), and such defects often lead to delayed union or nonunion during rehabilitation. An ideal porous scaffold should possess adjustable porosity and excellent interconnectivity to facilitate cell infiltration and proliferation (Shen et al., 2021; Li Z. et al., 2024). Consequently, systematic and in-depth research on the structural and functional properties of porous bone scaffolds is urgently required to meet the individualized treatment demands of patients with large segmental bone defects and promote the development of bone repair therapeutic techniques.

Native bone possesses a radially graded porous structure: the inner region has high porosity, which reduces weight and facilitates nutrient transport, while the outer region has low porosity to bear mechanical loads (Maconachie et al., 2019). From the macroscopic to the nanoscale, native bone tissue exhibits at least 12 levels of fractal-like hierarchical structures (Reznikov et al., 2018). Trabecular (cancellous) bone contains abundant fractal foam structures, with a porosity ranging from 50% to 90% (Currey, 2013; Reznikov et al., 2014; Wu et al., 2018; Vafaeefar et al., 2023). Bone scaffolds are employed for bone tissue replacement, with the primary aim of ensuring adequate stability at the surgical site, promoting bone tissue regeneration (Wu et al., 2021), and ultimately achieving integration with native tissue (Ghelich et al., 2022). The development of bone tissue scaffolds is typically categorized into two approaches: mechanical performance-driven and bone ingrowth-driven (Lv et al., 2024). In the mechanical performance-driven design, the primary goal is to achieve the effective modulus, strength, and permeability required for bone scaffolds (Jiao et al., 2022; Uklejewski et al., 2024). For instance, Uth et al. (Uth et al., 2017) developed a synthetic scaffold composed of type I collagen and nano-hydroxyapatite, which was engineered to maximize the compressive modulus. Poh et al. (Poh et al., 2019) adopted a density-based topology optimization method to regulate the porosity distribution of the scaffold under stiffness constraints. Wang et al. (Wang et al., 2025) proposed a novel data-driven reverse design framework for spinodoid bone scaffolds, and the results showed that the elastic constant and anisotropy of the novel bone scaffold highly matched the elastic constant of the target bone tissue. Biomimetic design of porous scaffolds is an emerging design paradigm that mimics the native bone system. It aims to achieve breakthroughs in the performance of artificial materials by replicating the structural features of native bone (Liu et al., 2023; Kai et al., 2025). By conducting biomimetic studies on the elastic properties of native bone, porous scaffolds can create a mechanical environment analogous to that of native bone under external physiological loads, thereby facilitating bone growth (Wu et al., 2014). Additionally, biomimetic design targeting the mechanical properties of native bone is conducive to reducing stress shielding, which in turn promotes bone ingrowth within the porous scaffolds (Al-Barqawi et al., 2022; Luo et al., 2017)—a feature particularly valuable for the repair and treatment of large segmental bone defects (Zhang et al., 2022). Furthermore, the adoption of biomimetic design principles constitutes an effective approach to enhancing the overall performance of porous scaffolds and optimizing their geometric structures (Liu et al., 2022; Zhang et al., 2024).

Fractal theory and its application in tissue engineering scaffolds offer a transformative conceptual framework for the design optimization (Liu et al., 2025). Qu et al. (Qu et al., 2021) proposed a radially layered gradient-controllable fractal porous scaffold via fractal design, which was subsequently fabricated using electron beam 3D (EB-3D) printing. Ullah et al. (Ullah et al., 2021) demonstrated the application of fractal geometry for simulating porous structures and subsequent fabrication via 3D printing technology. Zhang et al. (Zhang et al., 2021) employed finite element simulation to explore the potential application of the Menger sponge fractal NiTi structure as a bone implant. Stanciu et al. (Stanciu et al., 2023; Stanciu et al., 2023) developed a computational model that leverages tree-like fractals and additive manufacturing (AM) techniques to enable the design of complex internal structures. Xiao et al. (Xiao et al., 2023) developed and validated a fractal-based mathematical model for capillary fluid transport in fibrous porous materials, aiming to unravel the fundamental physical mechanisms governing fluid flow behavior in rough-walled porous media. Bigoni et al. (Bigoni et al., 2020) revealed that the porosity of biomorphic apatite exhibits lacunar fractality, which exerts a profound influence on fracture propagation and trajectory. Jian et al. (Jian et al., 2023) extracted the physical fractal space of bone fibers based on the micro-nano composite structure of cortical bone and derived fractal operators. Li S. et al. (2024) formulated a novel theoretical model comprising spherical particulate units and stochastically distributed dendritic branching architectures, which was established based on fundamental fractal geometry principles. Tu et al. (Tu et al., 2024) established an effective permeability model for fractal tree-like branching networks and examined the effects of structural parameters on the seepage process of such networks. Stanciu Birlescu et al. (Stanciu Birlescu et al., 2025) proposed a polynomial regression model based on fractal geometry to predict variations in mechanical response, which can be employed to forecast the uniform deformation of patterns. Although tree-like fractal structures can be considered for the internal design of AM-fabricated components, research in this area remains in its infancy (Stanciu Birlescu et al., 2023). Furthermore, whether tree-like fractals possess superior mechanical properties remains a topic requiring further investigation. Consequently, more comprehensive systematic studies are warranted on the structural design principles and mechanical properties of dendritic fractal-based bionic porous scaffolds.

Inspired by the branching morphology of natural trees, a design paradigm for porous bone scaffolds mimicking tree-like structures was proposed. A comprehensive workflow encompassing biomimetic design and 3D printing fabrication of porous scaffolds was established. By optimizing fractal parameters, trabecular diameter, and related structural parameters, personalized porous scaffolds with radial gradient of porosity can be rationally designed. Four distinct fractal-structured porous scaffolds were designed and fabricated via selective laser melting (SLM) 3D printing using Ti6Al4V alloy. Subsequently, the porosity distribution profiles, mechanical response characteristics, and damage modes and failure mechanisms of the as-prepared scaffolds were systematically investigated. Furthermore, compression tests were conducted to explore the damage behavior of both the biomimetic porous scaffolds and natural cortical bones. This study furnishes theoretical support and practical guidance for the design of radial gradient-tunable biomimetic bone scaffolds.

## 2 Materials and methods

Given the interpatient individual differences and their personalized demands for large-segment bone implants, systematic investigations into the parametric design, mechanical properties, failure modes, and fabrication processes of bone repair implants can facilitate the development of implants with structure and performance more closely matching those of native bone. Bone trabeculae and natural trees share analogous branching architectures, with highly similar morphological distributions (Figure 1). Building upon this insight, a design framework for biomimetic bone scaffolds featuring tree-like fractal architectures was established, and based on this framework, biomimetic porous scaffold models with distinct fractal parameters were developed.

**FIGURE 1:**
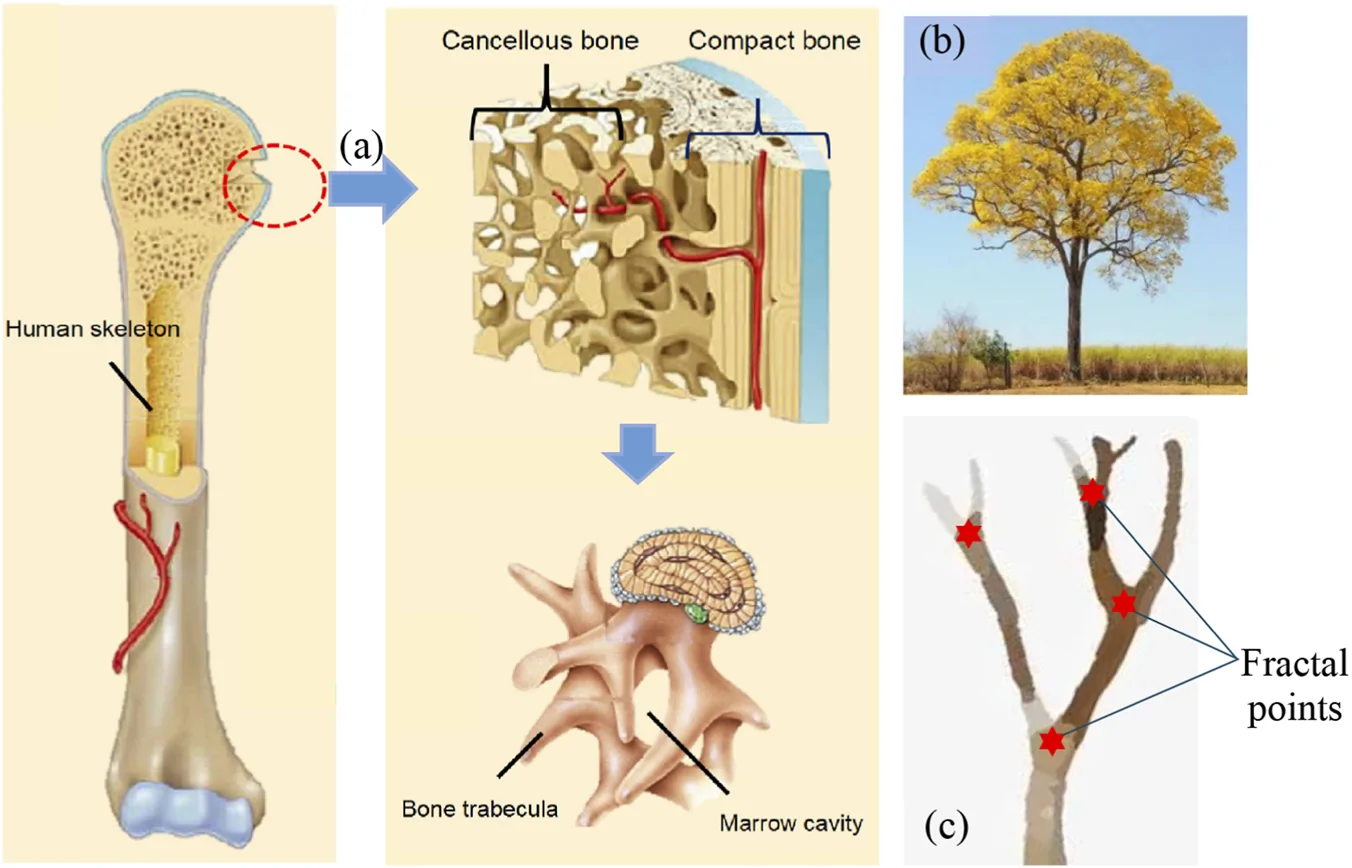
The branching morphology of trabecular bones and natural trees. **(a)** The branching characteristics of trabecular bone, **(b)** the overall morphology of the tree, **(c)** branch structure and corresponding branching nodes.

### 2.1 Tree-like fractal design theory of bone scaffold

Natural tree branches may appear disordered and erratic, yet they adhere to distinct growth laws. Essentially, a tree consists of roots, a trunk, and a crown, as illustrated in (Figure 1b). The tree crown consists of primary and lateral branches with an outward-spreading configuration (Figure 1c). presents the junction node at branch bifurcations, which is defined as the fractal point.

Theoretical analysis of bionic porous scaffold design inspired by dendritic fractal structure.

1. General assumption
  The radial cross-sectional morphology of the trabecular bone is roughly cylindrical, as illustrated in (Figure 1a) (Wang et al., 2016). Based on this morphological feature, a tree-like fractal structure was constructed along the radial direction of bone, as illustrated in (Figure 2). The following assumptions were formulated for the theoretical analysis of dendritic fractal structure scaffolds: (a) The trabecular is a regular cylinder, and at the same order, the cross-sectional diameter of the bone trabecular is equal. (b) The fractal branching angles remain consistent among trabecular units of identical hierarchical order. For bifurcating trabeculae, the angle bisector of the two branches coincides with the axis of the preceding order trabeculae. (c) The morphological geometric parameters of subsequent hierarchical trabecular structures scale proportionally with those of their prior-order counterparts. (d) The osseous matrix lamellae and trabecular bone structures exhibit an alternating stacking distribution along the axial orientation; the bone matrix exhibits a concentric circular configuration (as illustrated in Figure 2b) with a circular cross-section.
2. Parameter characterization
  a. Length (*l*_0_) and diameter (*d*_0_) of primary trabeculae: *l*_0_ is the length of the primary trabeculae, *d*_0_ is the diameter of the primary trabeculae. *l*_1_, *l*_2_, *l*_3_. are the lengths of the secondary fractal bone trabeculae, *d*_1_, *d*_2_, *d*_3_ … are the diameters of the secondary fractal bone trabeculae.
  b. Branching angle (*θ*) of bone trabeculae: The fractal branching angle is defined as the deflection degree of subordinate trabecular branches away from the central axis of upper-level trabecular structures. The fractal angles of each order are denoted as *θ*_1_, *θ*_1_’, *θ*_2_, *θ*_2_’, *θ*_3_ … (as illustrated in Figure 2a).
  c. Fractal order (*n*): The fractal order refers to the level of trabecular bifurcation. When there are no fractal points or bifurcated trabeculae, *n* = 0.
  d. Number of bifurcations (*m*): The number of trabecular bifurcations refers to the number of trabeculae generated from a single preceding-order trabecula.
  e. Ratio factors (*γ*, *μ*): *γ* represents the length ratio of secondary order trabeculae to superior trabeculae, and *μ* denotes the diameter ratio between secondary trabeculae and superior trabeculae. The relevant calculation formulas are shown below:
$$\gamma =\frac{l_{j}}{l_{j-1}},\mu =\frac{d_{j}}{d_{j-1}},j=1,2,3,\cdots ,n$$ (1)
  where *l*_*j*_ and *l*_*j*-1_ denote the lengths of trabeculae at the *j*th and (*j*-1)^th^ hierarchical orders, *d*_*j*_ and *d*_*j*-1_ correspond to their respective cross-sectional diameters.
  f. Bone matrix structural parameters: The number of circular bone matrix rings is denoted as N, and each ring has a circular cross-section (as illustrated in Figure 2b). *r*_1_,*r*_2_,*r*_3_ … *r*_N_ are the radii of each bone matrix ring, where *r*_1_ is the radius of the innermost bone matrix ring and *r*_N_ is the radius of the outermost bone matrix ring. The bone matrix volume *V*_*m*_ is calculated as follows:

**FIGURE 2:**
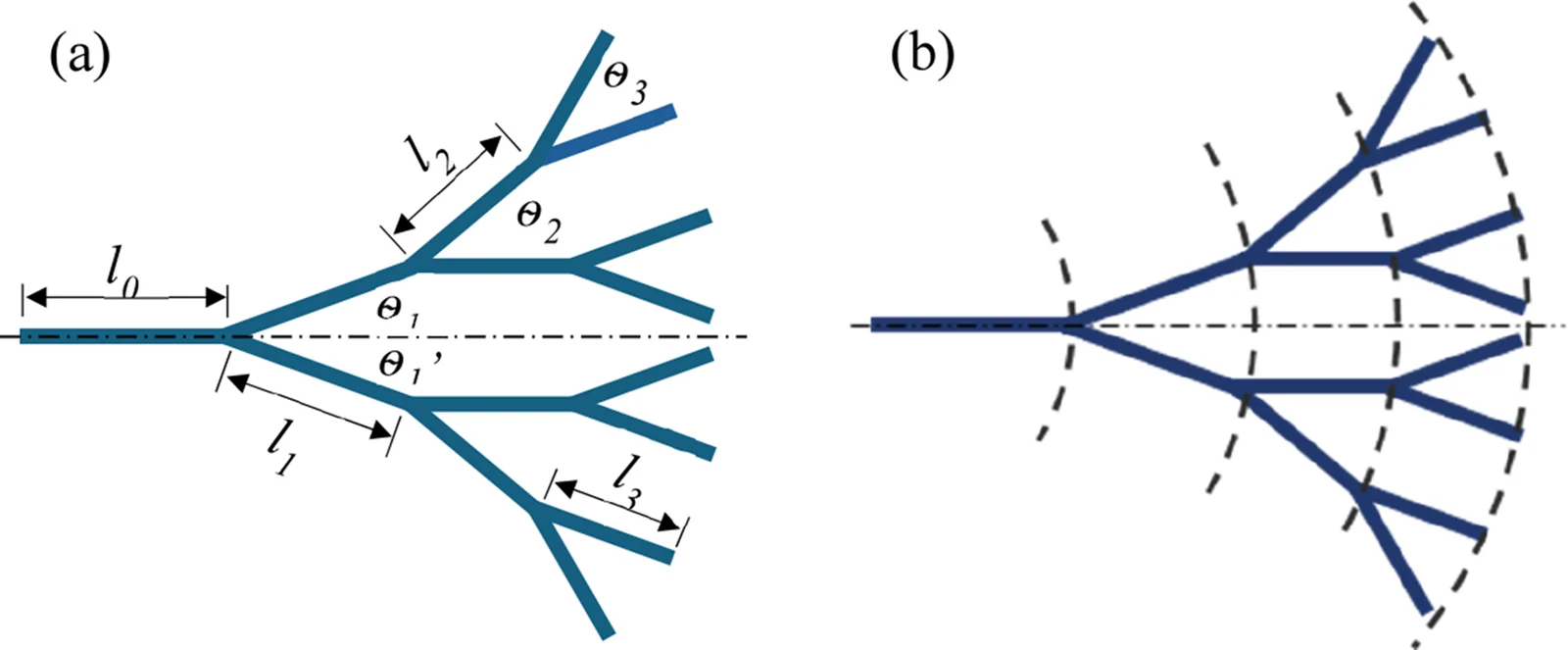
Schematic illustration of the radial fractal architecture of trabecular bone. **(a)** Third-order fractal structure, **(b)** third-order trabecular and circumferential bone matrix structure.

$$V_{m}=\sum_{i=1}^{N}V_{mi}=\sum_{i=1}^{N}2\pi^{2}R_{i}\cdot r_{i}^{2},i=1,2,3,\cdots ,N$$ (2)

where *R*_*i*_ is defined as the spacing between the central axis of the *i*th bone matrix ring and the geometric center of the biomimetic bone scaffold.

According to the above equation, when the cross-section of the trabecular bone is circular, the volume of the bone matrix is calculated as follows:

$$V_{bc}=\sum_{j=0}^{n}V_{jbc}=\sum_{j=0}^{n}m_{j}\pi {\left(\frac{d_{j}}{2}\right)}^{2}l_{j}=\frac{\pi }{4}d_{0}^{2}l_{0}\frac{1‐{\left(m\gamma \mu \right)}^{n+1}}{1‐m\gamma \mu }$$ (3)

where *m*_*j*_ represents the bifurcation quantity of the bone trabeculae in the *j*th fractal hierarchy, *d*_*j*_ represents the cross-sectional diameter of the trabeculae in the *j*th fractal, and *l*_*j*_ represents axial length of trabecular structures in the *j*th fractal.

Similarly, the network volume of bone trabeculae with elliptical cross-sections is calculated as follows:

$$V_{be}=\sum_{j=0}^{n}V_{jbe}=\sum_{j=0}^{n}m_{j}\pi {\frac{A_{ej}}{2}\cdot \frac{B_{ej}}{2}l}_{j}=\frac{\pi }{4}A_{e0}{B_{e0}l}_{0}\frac{1‐{\left(m\gamma \alpha \beta \right)}^{n+1}}{1‐m\gamma \alpha \beta }$$ (4)

where *A*_*ej*_,*B*_*ej*_ and *l*_*j*_ denote the short axis, long axis, and length of the *j*th order fractal elliptical bone trabeculae, respectively. *α* and *β* represent the short-axis ratio and long-axis ratio of elliptical bone trabeculae between the *j*th and (*j*-1)-th order fractals, respectively.

Based on the total volume of the trabecular network, the porosity of the entire biomimetic porous scaffold can be calculated via the formula *k* = 1- *xV*_*b*_ -*V*_*m*_, where *x* denotes the number of circular arrays composed of tree-like fractal monomers. In addition, along the radial direction of the biomimetic bone scaffold, the porosity within each circular ring defined by two adjacent fractal points can be computed individually.

By adding the lengths of all fractal order beams, the overall length *L*_*bt*_ can be obtained, which is expressed as follows:

$$L_{bt}=\sum_{j=0}^{n}l_{j}=l_{0}\frac{1-\gamma^{n+1}}{1-\gamma }$$ (5)

Based on Equations 3–5, the volume and length of tree-like fractal bone trabecular monomers can be calculated. The entire trabecular network can be equivalent to a curved bone trabecula with a total length of *L*_*bt*_ and an equivalent cross-sectional area of *S*. For bone trabeculae with circular or elliptical cross-sections, the equivalent cross-sectional areas of the tree-like fractal bone trabecular network—denoted as *S*_*bc*_ for circular cross-sections and *S*_*be*_ for elliptical ones—are given by:

$${S_{bc}=V}_{bc}/L_{bt}=\frac{\pi }{4}d_{0}^{2}\frac{1-\gamma }{1-\gamma^{n+1}}\frac{1-{\left(m\gamma \mu \right)}^{n+1}}{1-m\gamma \mu }$$ (6)

$${S_{be}=V}_{be}/L_{bt}=\frac{\pi }{4}{A_{e0}B}_{e0}\frac{1-\gamma_{e}}{1-\gamma_{e}^{n+1}}\frac{1-{\left(m\gamma \alpha \beta \right)}^{n+1}}{1-m\gamma \alpha \beta }$$ (7)

### 2.2 Design process of bionic porous scaffold

Leveraging the fractal theory established in this study, porous scaffolds with radial gradient features and diversified structural performances can be effectively constructed. Through optimizing key parameters—including bifurcation number *m*, fractal angle *θ*, fractal order *n*, and branching trabecular diameter—personalized radial pore gradient porous scaffolds can be constructed. Furthermore, the structural characteristics and porosity distribution of the scaffolds are directly governed by fractal curve morphology of the trabeculae and the quantity of annular bone matrix layers. According to the location and size of clinical large bone defects, a second-order fractal topology was adopted to develop a radial gradient bionic scaffold for targeted case verification. Specific workflow is illustrated below (Figure 3).

**FIGURE 3:**
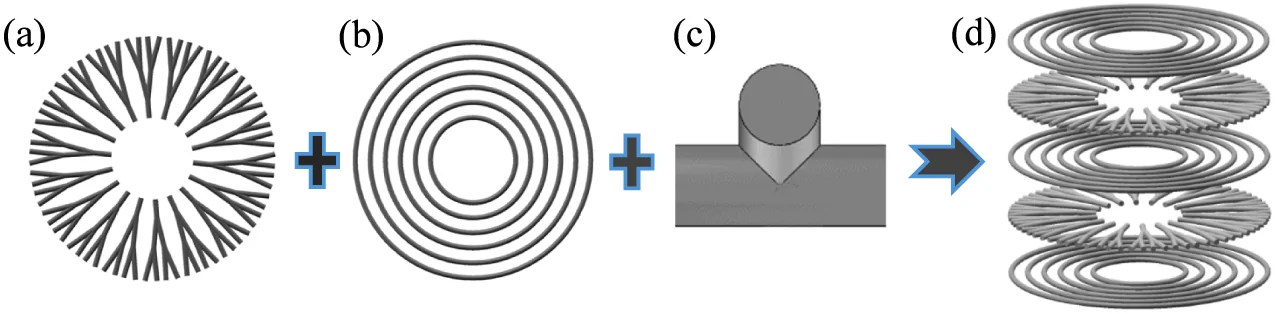
Parametric design process of radial gradient biomimetic scaffolds. **(a)** Tree-like fractal structure layer, **(b)** concentric annular bone matrix layer, **(c)** connection structure between the tree-like fractal layer and the bone matrix layer, **(d)** bottom-up stacking order of biomimetic scaffold.

First, reconstruct a model of healthy bone tissue in patients through CT scanning, the dimensions of the target repair area—specifically its internal diameter, external diameter, length, and porosity distribution—are determined via left-to-right symmetry analysis.

Second, based on the pore characteristics of defective bone tissue, a tree-like fractal unit is designed based on the optimized fractal parameters and fractal curve morphology; subsequently, a fractal layer is constructed by arranging the fractal units in a circumferential array (as illustrated in Figure 3a).

Third, the diameter and quantity of the concentric rings are determined based on design calculations, and the concentric rings are fabricated to form the bone matrix structure (as illustrated in Figure 3d).

Fourth, the connection characteristics at the fractal intersection points are designed, and the bone matrix layers and tree-like fractal layers are connected via cylindrical connectors with diameters matching those of the concentric rings (as illustrated in Figure 3c).

Fifth, based on the target porosity and radial distribution profile, the key fractal parameters and fractal curve morphology are optimized. For the quantitative characterization of scaffold radial porosity distribution, five uniform regional sections were delineated along the radial orientation, with markings a, b, c, d, and e arranged in an interior-to-exterior sequence.

Sixth, lamellar units with fractal features and bone matrix substances are piled alternately in the axial spatial orientation; the interlayer contact areas are connected via the connection characteristics designed in the third step, thereby constructing a 3D model of the tree-like fractal porous scaffold (as illustrated in Figure 3d).

Seventh, comprehensive performance evaluation is implemented for the prefabricated porous scaffold, and further optimization of key fractal parameters and porosity is conducted.

Eighth, Eighthly, comprehensive comparisons of geometric profiles, porosity, permeability and mechanical properties are implemented between the biomimetic scaffold and native bone; based on the observed differences, the design parameters in the previous steps can be optimized to meet the personalized needs of patients.

Finally, the porous scaffold is 3D-printed via the SLM additive manufacturing process.

Based on the aforementioned parameterized design process, four biomimetic bone scaffold models with distinct fractal orders were developed, where the fractal order *n* was set to 0, 1, 2, and 3, respectively. The number of concentric rings in the bone matrix was set to six; other key parameters included a bone trabecular diameter of 0.5 mm, an outer diameter *D* of 24 mm, and an inner diameter

*d* of 8 mm (as illustrated in Figure 4).

**FIGURE 4:**
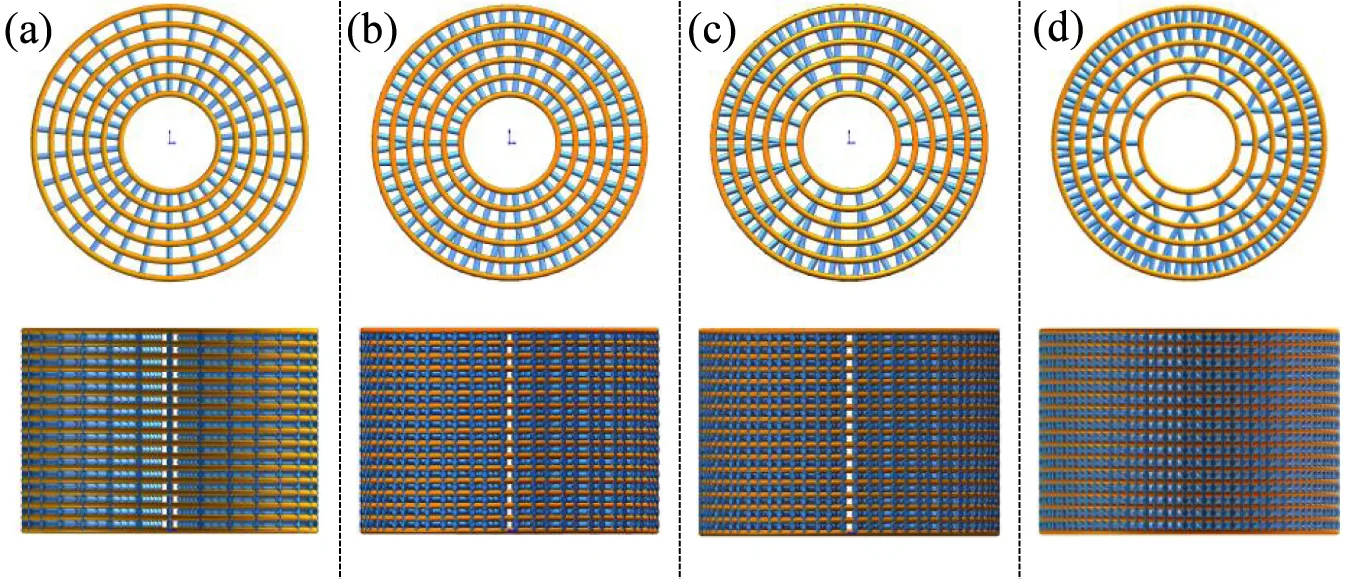
Tree-like fractal structure porous bone scaffold model. **(a)** Axial and radial views of the zero-order model, **(b)** axial and radial views of first-order model, **(c)** axial and radial views of second-order model, **(d)** axial and radial views of third-order model.

### 2.3 Additive manufacturing of biomimetic bone

To verify the manufacturability of the designed biomimetic porous scaffolds, four sets of tree-like fractal biomimetic scaffolds with distinct fractal orders were fabricated via SLM additive manufacturing technology. Ti6Al4V powder with uniform spherical morphology was utilized as the substrate material. The powder exhibited a dimensional distribution of 15–45 μm and Hall flow speed is 45S, and the SLM manufacturing process was implemented on a BLT-S210 3D printer (Xi’an Bright Laser Technologies Co., Ltd.). The 3D printer equipped with a 500 W high-power laser and a focused beam spot of 60 μm. The processing parameters adopted in this study were as follows: laser power of 175 W, scanning speed of 800 mm/s, hatch spacing of 70μm, layer thickness of 30μm, and laser spot size of 60 μm. Subsequently, the fabricated samples were detached from the substrate via wire electrical discharge machining. The fabricated biomimetic porous scaffold specimens underwent ultrasonic cleansing using absolute ethanol, aiming to eliminate residual unmolten particles attached to the outer surface.

### 2.4 Mechanical properties experiment

To investigate the mechanical behavior and failure modes of biomimetic porous scaffolds, quasi-static compression tests were performed on the 3D-printed samples using an electronic universal testing machine (Model: DF13.105T; Manufacturer: Sinotest Equipment Co., Ltd.) (Figure 5a). The boundary conditions were strictly defined to ensure test accuracy and reproducibility. The bottom surface of each specimen was fully fixed on the lower rigid platen, and the specimen was aligned concentrically with the loading axis. The upper compression platen was restricted to uniaxial translation along the vertical (height, *Z*-axis) direction, with all other degrees of freedom locked. To minimize end-friction interference, both end faces of the specimens were polished to a parallelism tolerance of ≤0.05 mm. The complete loading protocol was implemented in accordance with the ISO 13314–2011. First, a preloading step was conducted at a rate of 0.2 mm/min up to a preload of 10 N and held for 10 s to eliminate initial gaps and ensure full contact between the specimen and platens. Subsequently, formal uniaxial compression was carried out at a constant crosshead speed of 0.5 mm/min (Xiong et al., 2020). The geometric dimensions of the specimens were as follows: a 24 mm outer diameter (*D*), an 8 mm inner diameter (*d*), and a 16.5 mm height (*h*). The test was automatically terminated when the compressive load dropped to 70% of the peak load or a penetrating macroscopic crack was observed on the specimen. Force and displacement data were synchronously acquired at a sampling frequency of 10 Hz, from which the corresponding stress–strain curves were derived. The Young’s modulus of each porous specimen was calculated from the slope of the linear elastic segment of the stress–strain curve.

**FIGURE 5:**
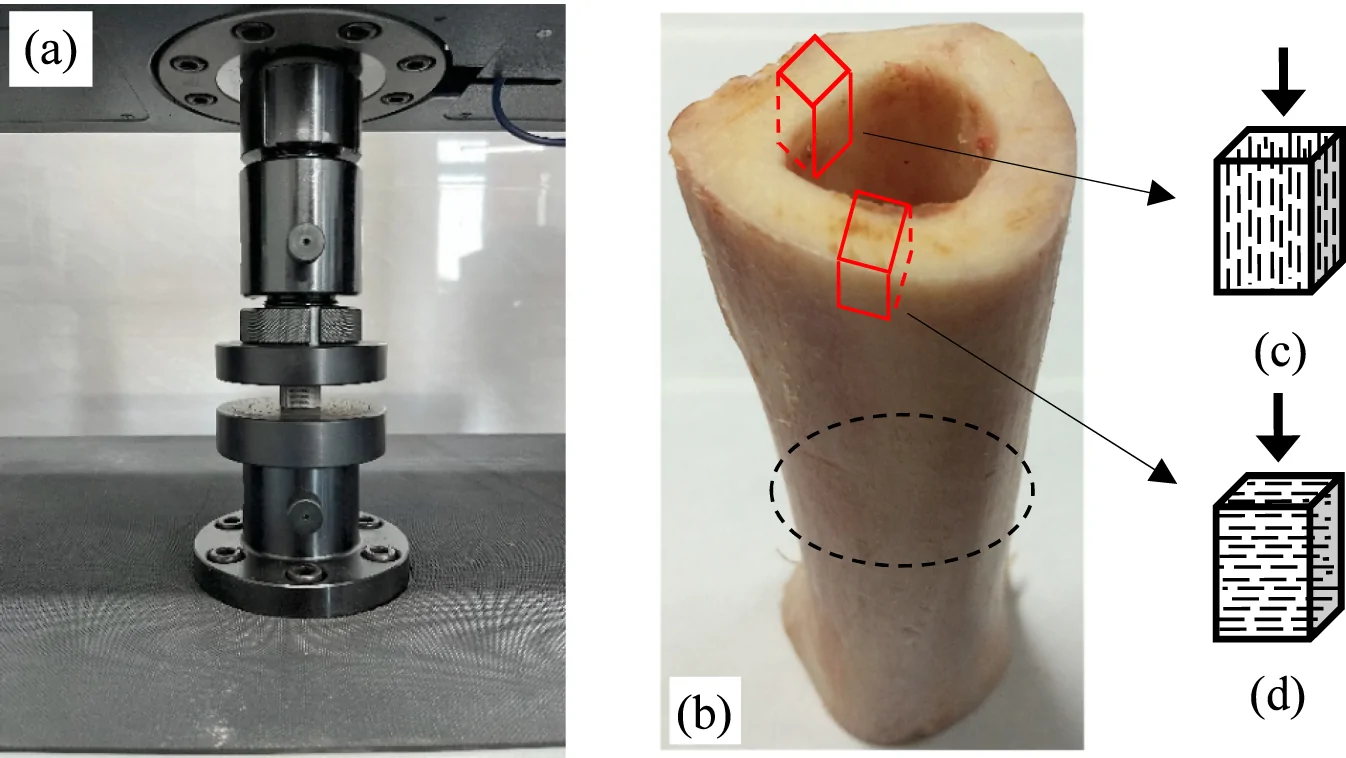
Compression test and bovine femur sampling. **(a)** 3D printed biomimetic bone compression test, **(b)** backbone of bovine femoral, **(c)** longitudinal specimen, **(d)** transverse specimen.

The elastic modulus and yield strength of porous scaffolds are structure-dependent macroscopic mechanical properties rather than intrinsic material attributes, and their values are strongly positively correlated with the relative density (or apparent mass) of the scaffolds. Direct comparison of absolute mechanical values among specimens with differing relative densities fails to identify the origin of performance differences; specifically, it cannot distinguish whether the discrepancy arises from the optimization of structural design itself or from density variations between specimens. Accordingly, to eliminate the interference of mass (i.e., relative density) differences among the four groups of biomimetic scaffolds on the cross-group comparison of mechanical properties, a dimensionless normalization procedure was applied to the mechanical parameters of all specimens. First, the compressive stress was normalized based on the raw data from compression tests. Subsequently, the normalized elastic modulus and normalized compressive strength were derived from the normalized stress–strain curves. The calculation formula for the normalized stress σ of each scaffold is defined as follows:

$$\sigma_{norm}=\frac{\sigma }{\rho_{r}}$$ (8)

where, *σ* is the experimentally measured stress (MPa); *ρ*_*r*_ denotes the relative density, *ρ*_*r*_ = *ρ* ^*^/*ρ*_*s*_, in which *ρ* ^*^ is the apparent density of biomimetic scaffolds (g/mm^3^), and *ρ*_*s*_ is the density of bulk Ti6Al4V alloy (g/mm^3^).

For comparative analysis of the failure characteristics between biomimetic bone scaffolds and native cortical bone, femoral cortical bone specimens from Chinese yellow cattle (18–24 months old) were selected for experimental research. First, a segment of the femur was sectioned using a CNC grinder (as illustrated in Figure 5b). Second, specimens were harvested along its longitudinal and transverse directions to obtain longitudinal specimens (the longitudinal direction of the specimen was aligned with the femoral axis) (Figure 5c) and transverse specimens (the transverse direction of the specimen was perpendicular to the femoral axis) (Figure 5d). The specimen dimensions were 6.5 mm × 4 mm × 4 mm. A total of 20 specimens were prepared, with 10 longitudinal specimens and 10 transverse specimens, respectively. Quasi-static compression tests were performed on each specimen at a loading rate of 0.5 mm/min. To maintain the moisture content of the bone tissue, a balanced salt solution was sprayed onto the femoral specimens during sample preparation; thereafter, the prepared specimens were soaked in the balanced salt solution. Prior to testing, the specimens were removed from the solution and air-dried. Subsequently, compression tests were carried out under identical equipment and boundary conditions as those adopted for the 3D-printed specimens. Post-test, the failure behaviors of the longitudinal and transverse specimens were compared and analyzed.

### 2.5 Finite element simulation

To predict the mechanical properties and stress distribution of the tree-like fractal porous scaffolds, finite element (FE) analysis was performed using Abaqus software (version 2021, Dassault Corp., USA). To determine the optimal mesh size, mesh convergence and mesh independence verification were conducted for each model. Taking the zero-order fractal model as an example, mesh optimization was performed, resulting in approximately 1,094 690 tetrahedral elements and a maximum mesh size of 0.2 mm. The bottom surface of the model was subjected to fixed constraints, restricting all six degrees of freedom. The upper pressure plate was constrained as a rigid body, and a uniform displacement load was applied along the negative Z-axis at a rate of 0.5 mm/min. The displacement load was set to 5% of the specimen height, corresponding to 0.83 mm. To compare and analyze the mechanical properties of each group of specimens, the same loading rate and boundary conditions were applied to the four groups of porous scaffolds. The material of the porous scaffold is Ti6Al4V, with an elastic modulus of 113.8GPa, Poisson’s ratio of 0.32, yield strength of 970MPa, and density of 4430 kg/m^3^ (Qin et al., 2022). The upper pressure plate was set to have an elastic modulus of 193GPa, a density of 7.93 g/cm^3^, and a Poisson’s ratio of 0.29.

Based on the stress distribution and stress values obtained from simulations, the elastic modulus was calculated using the following equation:

$$E=\frac{Fh}{A\Delta h}$$ (9)

where *F* denotes the applied force (unit: N); *E* denotes the elastic modulus (unit: GPa); *h* denotes the specimen height (unit: m); *A* denotes the cross-sectional area of the biomimetic bone scaffold (unit: m^2^); and Δ*h* denotes the distance of movement (unit: m) (Ma et al., 2020).

## 3 Results and discussion

### 3.1 Parametric effects on fractal morphology

For investigating impacts of fractal factors on morphological features of fractal trabecular network architectures, the tree-like fractal morphology of such networks was analyzed using schematic diagrams under different fractal orders *n* and fractal angles *θ* (with circular bone trabecular cross-sections). Specifically (Figure 6), illustrates the morphology under different fractal orders *n*, and (Figure 7) depicts that under different fractal angles *θ*. Analysis of the fractal morphologies in these two figures reveals that higher values of fractal order and fractal angle lead to progressive complication of tree-like trabecular morphology. These findings demonstrate that key parameters of fractal architecture, including fractal order *n* and fractal angle *θ*, markedly modulate the morphological characteristics of trabecular networks. Accordingly, in clinical applications, personalized porous scaffolds can be designed by adjusting the geometric dimensions and fractal characteristics of the trabecular structure based on the morphological characteristics of patients’ osseous defect region, which opens up broad prospects for the customized design of bone implants.

**FIGURE 6:**
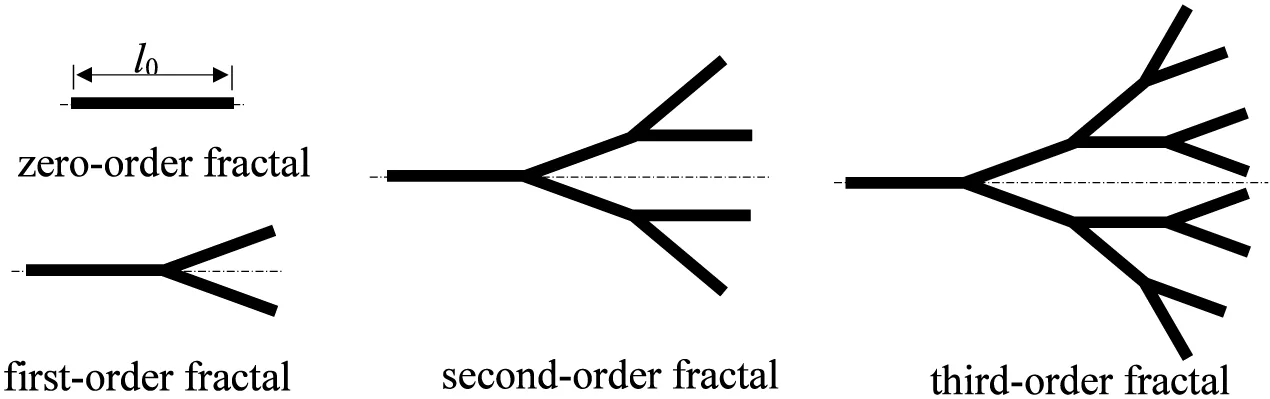
Tree-like fractal morphology of bone trabeculae with different fractal orders *n* (branching number *m* = 2, fractal angle *θ* = 20°). Zero-order fractal.

**FIGURE 7:**
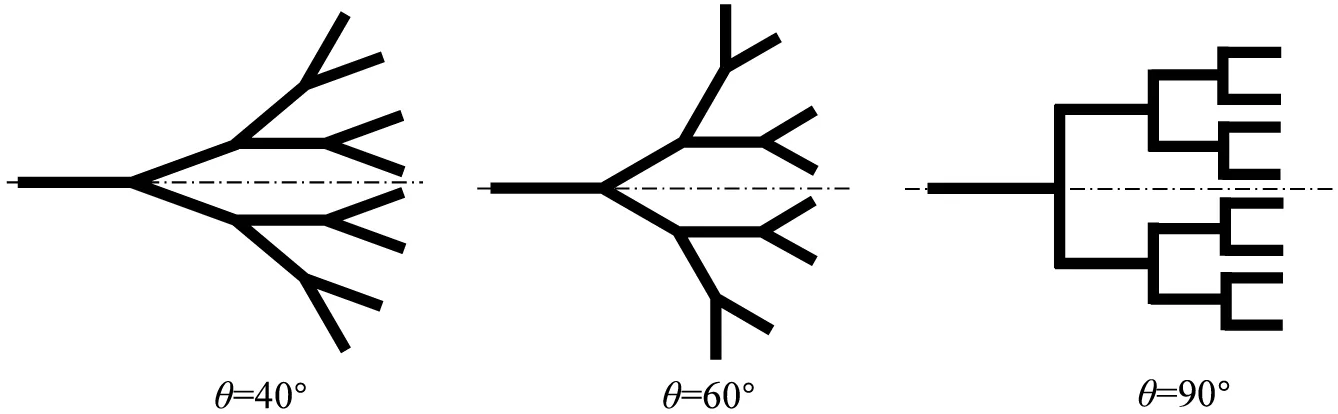
Fractal patterns of trabecular bone under various fractal angles *θ* (branching number *m* = 2, branching order *n* = 3).

### 3.2 Parametric effects on scaffold porosity

According to the established biomimetic porous scaffold models, the porosity of the five radial regions was calculated for each scaffold, with the corresponding radial porosity distribution profiles presented in (Figure 8). In addition, owing to the lack of bone trabecular distribution in the central area of the femur, this region exhibits a porosity of 100%. Analysis of the local porosity distributions of the biomimetic bone scaffolds reveals that key structural parameters, including fractal order *n* and fractal angle *θ*, exert a direct influence on the radial porosity distribution of the scaffolds. Specifically, Notably, second- and third-order fractal configurations possess elevated inner porosity and reduced outer porosity, which is consistent with the structural characteristics of native bone tissues. Furthermore, the second- and third-order fractal structures possess inner-zone porosities of 74% and 77%, respectively, whereas their outer-zone porosities are 67% and 63%, respectively. The analytical results demonstrate that the radial porosity gradient of the scaffold is substantially strengthened with the elevation of fractal order *n*. Thus, based on the porosity characteristics of the patient’s defect bone, optimization of key structural parameters enable the design of porous bone scaffolds with tailored structural characteristics, which can better cater to the personalized requirements of patients with bone defects.

**FIGURE 8:**
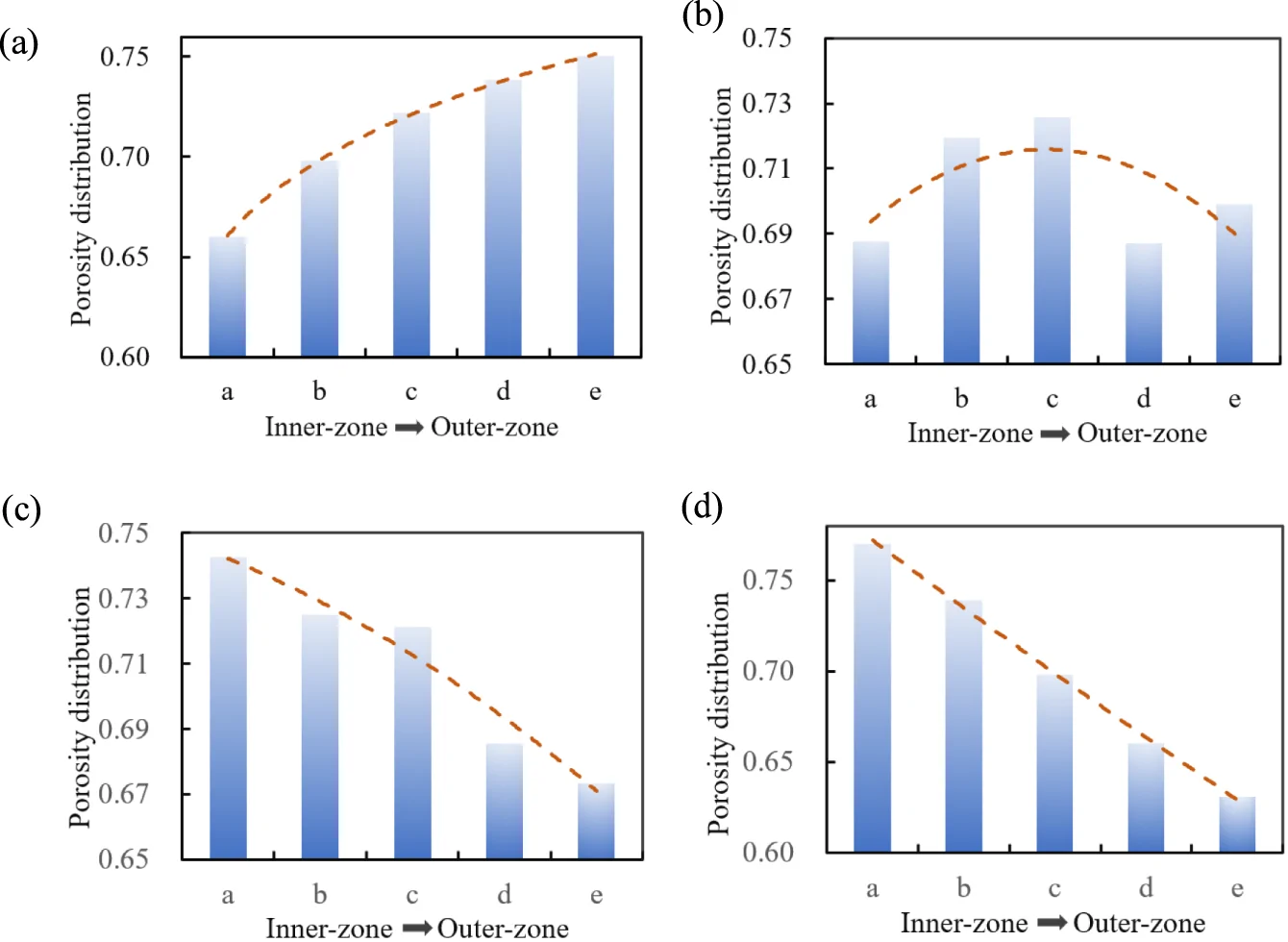
Porosity distribution along the radial direction of biomimetic fractal scaffolds. **(a)** Zero-order structure, **(b)** first-order structure, **(c)** second-order structure, **(d)** third-order structure.

The porous scaffold designed in this study features high porosity in its inner zone, mimicking the structure of trabecular bone and thus ensuring ample space for cell survival, proliferation, and nutrient transport. In comparison, the outer domain of the scaffold possesses a dense configuration with reduced porosity. This unique structural characteristic replicates the biomechanical morphology of cortical bone, enabling sufficient mechanical stabilization for tissue reconstruction. The radially gradient porosity distribution that gradually declines from its interior structure to the peripheral region of the scaffold is well consistent with the research results reported in the literature (Qu et al., 2021). Therefore, by optimizing fractal and branching structural parameters, personalized porous scaffolds can be tailored, thereby offering immense potential for their parametric design.

### 3.3 Morphological analysis of 3D printed specimens

The SLM fabricated specimens of tree-like biomimetic porous scaffolds are shown in (Figure 9). Dimensional measurement and analysis results reveal that the dimensions of the 3D-printed specimens are consistent with the designed model dimensions, and no obvious un-melted powder was observed on the surfaces. The biomimetic porous scaffolds exhibit a continuous radial gradient distribution of porosity, with no structural deformation detected (Figures 9b,d,g, and j). The pores in the vicinity of the porous scaffolds are clearly distinguishable, showing no blockage or collapse (Figures 9a,f,i and l). Specifically, the minimum peripheral pore size of the third-order fractal scaffold is 0.21 mm, while that of the second-order scaffold is 0.37 mm, the interlayer spacing of all four porous scaffolds is 0.5 mm. Such pore sizes are conducive to facilitating bone tissue ingrowth into the scaffolds (Ataee et al., 2018). Further analysis on the surface machining quality of fabricated specimens reveals that there is an obvious deviation in surface roughness between actual samples and the designed model shown in (Figure 4). The main factors affecting the surface quality are STL model accuracy, wire cutting machining accuracy and SLM forming accuracy. The process feasibility of the designed fractal bionic scaffolds was validated through specimen fabrication and geometric dimensional characterization. This provides a foundational guarantee for the practical deployment of bionic fractal scaffold constructs in bone tissue regeneration engineering.

**FIGURE 9:**
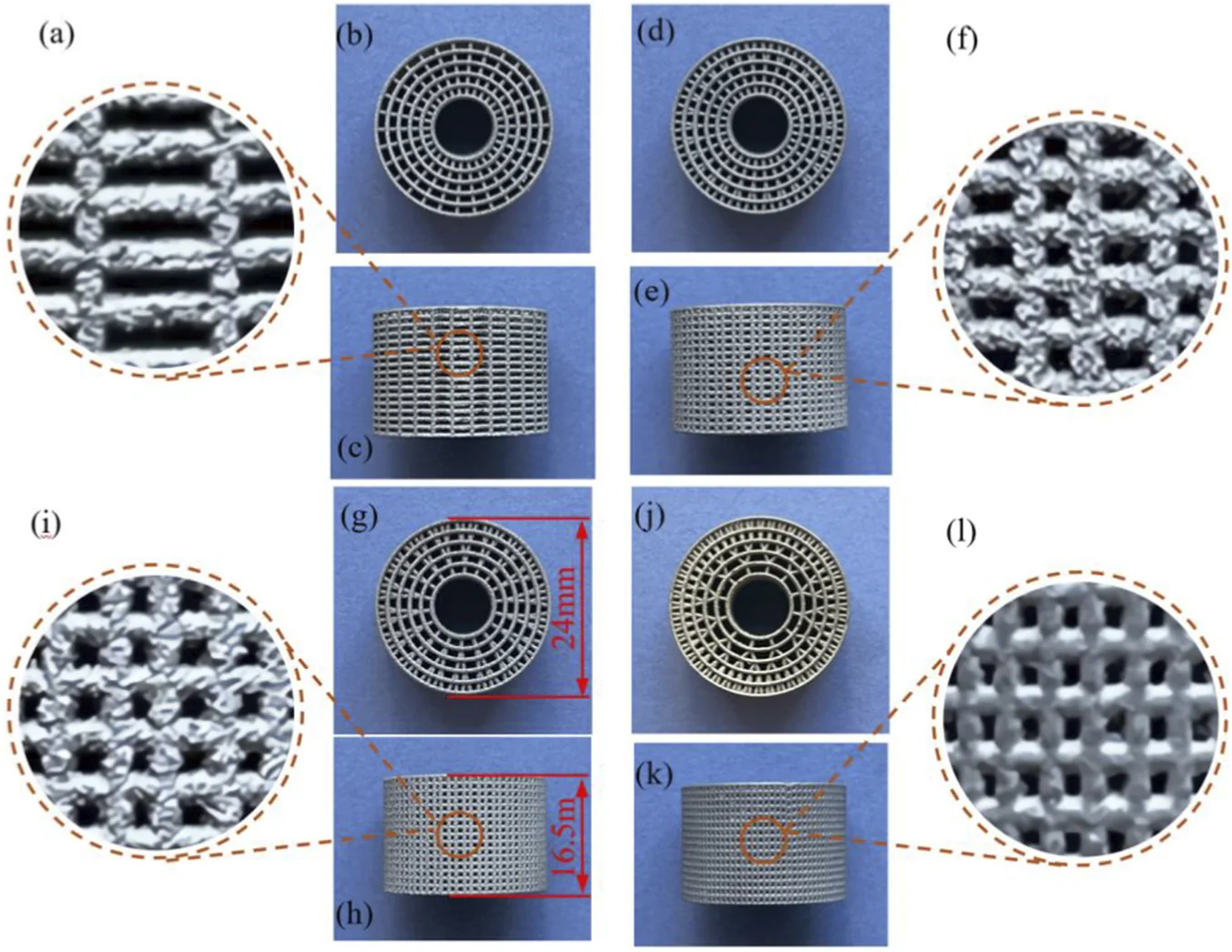
Images of SLM fabricated specimens of bionic porous scaffolds. Radial **(a)**, axial **(b)**, and microstructure **(c)** views of zero-order structure specimen; radial **(d)**, axial **(e)**, and microstructure **(f)** views of first-order structure specimen; radial **(g)**, axial **(h)**, and microstructure **(i)** views of second-order structure specimen; and radial **(j)**, axial **(k)**, and microstructure **(l)** views of third-order structure specimen.

### 3.4 Damage modes of biomimetic scaffold and natural femur

Quasi-static compression tests were conducted on the four types of biomimetic scaffold specimens and bovine femoral cortical bone specimens in accordance with ISO 13314–2011. Considering potential fabrication errors during SLM additive manufacturing, five scaffolds were selected per group. To compare and analyze the failure modes of biomimetic porous scaffolds and natural bone, 10 femoral cortical bone specimens were tested, comprising five longitudinal specimens and five transverse specimens. The typical failure modes of the biomimetic bone scaffolds and natural bovine femoral cortical bone are illustrated in (Figure 10). The fracture path of the zero-order fractal scaffold specimen is approximately X-shaped, with a prominent radial bulge observed in the middle of the specimen (Figure 10a). This failure mechanism is primarily attributed to its porosity distribution pattern, with comparatively lower porosity inside the structure and distinctly higher porosity in outer regions. In contrast, the fracture paths of the other three groups of scaffold specimens are oriented at approximately 45° relative to the specimen axis (Figures 10b–d). These failure modes are consistent with those reported for radially graded TMSP porous scaffolds (Lv et al., 2022). (Figure 10e) illustrate the fracture paths of longitudinal femoral cortical bone specimens under compression. Based on the failure mode, the fracture path of the longitudinal specimens forms an angle of approximately 0° with the loading direction, indicating that the longitudinal specimen fracture nearly along the axial direction of the femur (Figure 10f). depicts the fracture path of the transverse specimens under compression. The fracture path of the transverse specimens forms an angle of approximately 45° with the loading direction, which means that the transverse specimen fractures nearly along the 45° direction relative to the femoral axis (Figure 5b).

**FIGURE 10:**
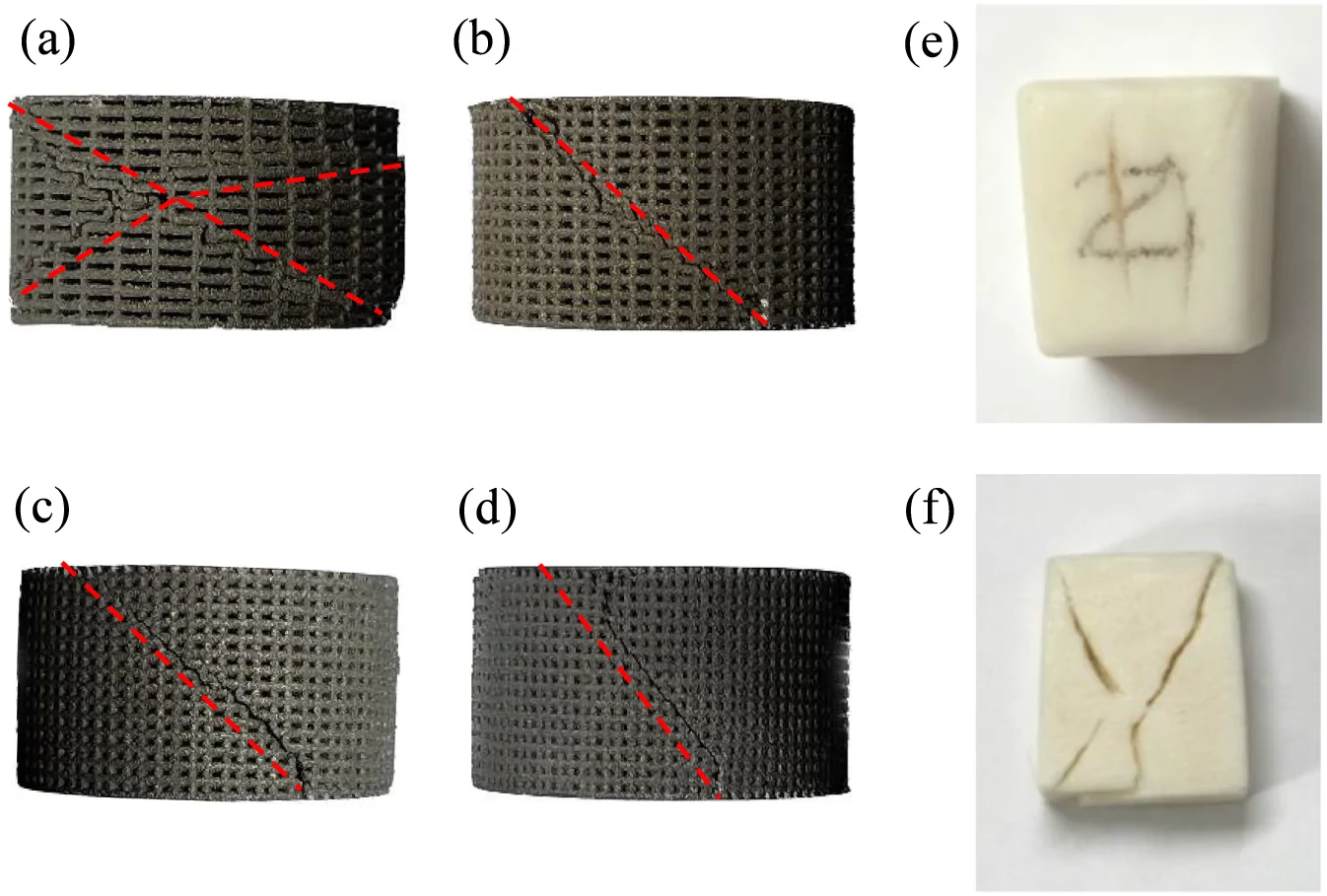
Damage modes of the tree-like fractal biomimetic bone scaffolds and natural cortical bone. **(a)** Zero-order fractal sample, **(b)** first-order fractal sample, **(c)** second-order fractal sample, **(d)** third-order fractal sample, **(e)** longitudinal cortical bone specimens, **(f)** transversal cortical bone specimens (The red dashed line represents the fracture path of the sample).

In addition, the transverse femoral cortical bone specimens also exhibited a V-shaped fracture under compressive loading. Comparative analysis of the failure modes between biomimetic porous scaffolds and natural femoral cortical bone reveals that the failure modes of bionic porous scaffolds are analogous to that of transversely compressed femoral cortical bone, but exhibits a notable discrepancy from that of longitudinally compressed femoral cortical bone. Owing to the staggered axial arrangement of bone matrix and bone trabecular fractal layers, the multilayered architecture of the biomimetic bone scaffolds can sustain greater axial loads. This effectively compensates for the inherent susceptibility of natural femurs to axial fracture under axial loading. Furthermore, the layered structure of the tree-like scaffolds enables a larger contact interface and more uniform stress distribution between the bone scaffolds and natural bone tissue. The stress distribution cloud maps of the four tree-like fractal biomimetic scaffold models under a 5% displacement load are illustrated in (Figure 11). Simulation analysis results reveal that the stress concentration points of all four models are primarily located at the fractal junctions and interlayer connections. Under the same compressive deformation, the maximum stress values of the four models differ significantly: the minimum maximum stress is observed in the zero-order biomimetic scaffold, while the maximum is in the third-order biomimetic scaffold. For third-order fractal architectures, the inner porous domain possesses higher porosity and contains fewer bone trabeculae. Such structural features diminish interlayer contact interfaces, which serves as the dominant cause of the above phenomenon. Comparison of the maximum stress of each model with the material’s yield stress reveals that all four models undergo yield deformation under the 5% displacement load. Furthermore, among the three fractal-containing models, the second-order fractal structure exhibits the smallest maximum stress and a more uniform stress distribution. Therefore, the stress distribution of the biomimetic bone scaffolds and the yield strength of the overall structure are significantly influenced by the fractal parameters of the tree-like fractal structure, adjusting these fractal parameters can thus tailor the mechanical properties of the biomimetic bone scaffolds.

**FIGURE 11:**
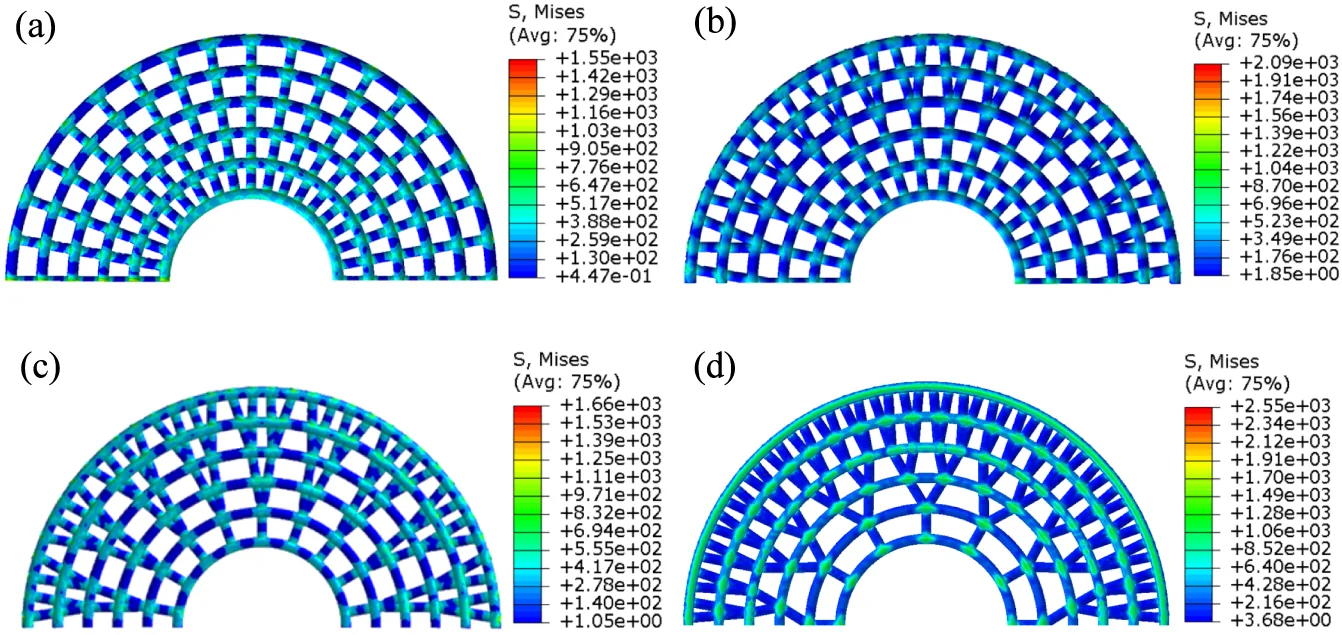
Stress distribution cloud maps of four biomimetic bone models. **(a)** Zero-order structure, **(b)** first-order structure model, **(c)** second-order structure model, **(d)** third-order structure model.

### 3.5 Mechanical properties of porous scaffolds

To visually compare the stress-strain mechanical characteristics of various fractal scaffolds, the mechanical test data of all porous scaffolds were normalized in this work (Figures 12a-d). present the normalized stress-strain response curves of bionic porous scaffolds with different fractal parameters, in which the brown curves denote the deviation of stress values. The stress-strain curves of the four fractal structures are highly consistent. Analysis of the experimental results reveals slight variations in mechanical responses among specimens with the same fractal parameters, with a variation of approximately 10% or less. These differences are primarily attributed to factors such as fabrication processes and experimental procedures. Further analysis of the experimental results for each group of specimens indicates that all specimens exhibit an elastic response in the initial stage of their stress-strain curves prior to reaching the yield point. The compressive failure process of Ti6Al4V tree-like fractal scaffolds can be primarily divided into four stages: (1) A weak nonlinear response can be observed ahead of linear elastic deformation, which is induced as the compression plate gradually achieves complete contact from the preliminary contact state with specimens. (2) In the linear elastic stage, strain energy is stored via the reversible deformation of the porous scaffold. (3) During the plastic deformation stage, a slight load increase induces substantial strain; as the strain increases, the bone trabeculae undergo mutual contact and gradually enters a densified state. (4) Fracture failure, which encompasses the entire process from crack initiation to complete structural collapse. The four fractal structures exhibit similar mechanical behaviors, with a notable distinction lying in their varying load-bearing resistance from the yield point to the fracture point; notably, no softening phenomenon was observed. After passing through the plastic region, the structure suddenly lost its load-bearing capacity, leading to complete failure. Furthermore, analysis of the yield points in the stress-strain responses of the four specimens indicates that the compressive strength of the scaffolds tends to increase as the fractal order increases. The third-order biomimetic specimens attain the maximum normalized compressive strength of 1051.81 MPa and the ultimate failure strain reaching 13%, as illustrated in (Figure 12).

**FIGURE 12:**
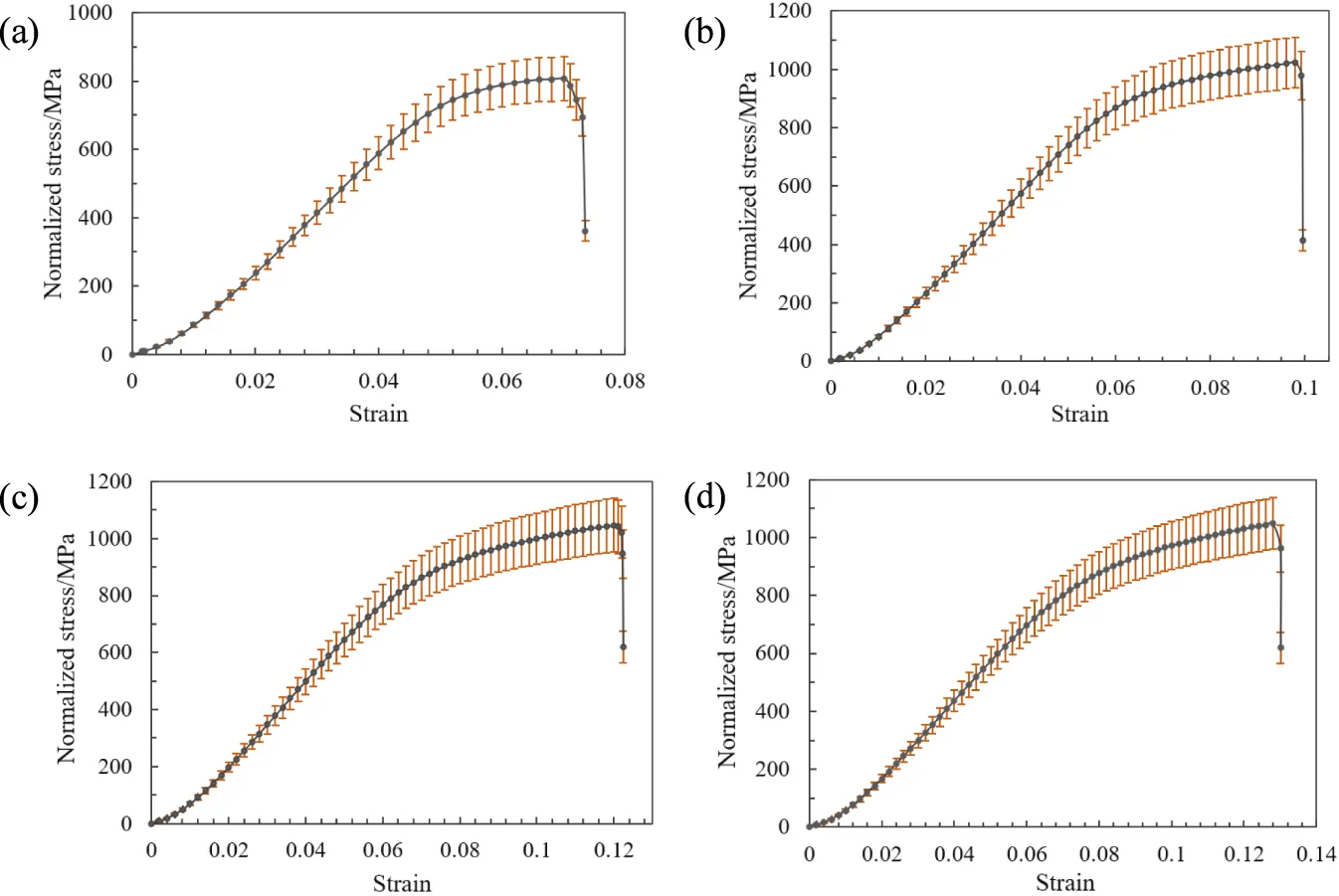
Normalized compressive stress-strain curves of four tree-like fractal biomimetic bone porous scaffolds. **(a)** Zero-order fractal model, **(b)** first-order fractal model, **(c)** second-order fractal model, **(d)** third-order fractal model.

The Young’s moduli of all the bionic samples, derived from compression tests and finite element (FE) calculations, are illustrated in (Figure 13). Specifically, the normalized Young’s moduli of the four fractal models are 15.078 GPa, 17.077 GPa, 15.325 GPa, and 13.602 GPa, respectively (The structural elastic models of the four biomimetic specimens are 5.093GPa, 6.048GPa, 5.14GPa, and 5.178GPa, respectively). The Young’s modulus of human cortical bone is approximately 15–25 GPa (Szabo et al., 2022; Zawadzki et al., 2022), whereas that of trabecular bone ranges from 0.1 to 4.5 GPa (Pak et al., 2025). The biomimetic scaffold designed in this study is developed for the repair of large-segment open-type bone defects and integrates the structural features of both trabecular and cortical bone. Accordingly, the overall elastic modulus of the as-proposed biomimetic scaffold falls within the modulus range of native bone tissue. Comparative analysis of experimental data indicates that elastic modulus values of zero-order and second-order specimens are roughly comparable. Among the four structural designs, the first-order specimen achieves the maximum elastic modulus, whereas the third-order counterpart presents the minimum value. The test results show favorable consistency with finite element simulation predictions. Both sets of data confirm that the first-order scaffold possesses the maximum elastic modulus, while the third-order scaffold obtains the minimum value. Furthermore, the FE simulation values are consistently higher than the experimental results, yet the two sets of data exhibit the same variation trend. This consistency indicates that the tree-like bionic specimens fabricated via SLM in this study possess stable mechanical properties. Integrated comparison of compressive strength (Figure 12) and elastic modulus (Figure 13) indicates that the third-order specimen exhibits optimal compression resistance yet maintains a relatively lower Young’s modulus. Accordingly, the tree-like fractal biomimetic bone developed in this work can attain integrated high strength combined with low modulus characteristics via rational optimization of characteristic parameters. Its mechanical characteristics show great similarity to native bone, this structure can substantially alleviate stress shielding effect and prolongs the service life of bone implants. This is consistent with the findings reported in relevant studies (Lv et al., 2022; Chen et al., 2019).

**FIGURE 13:**
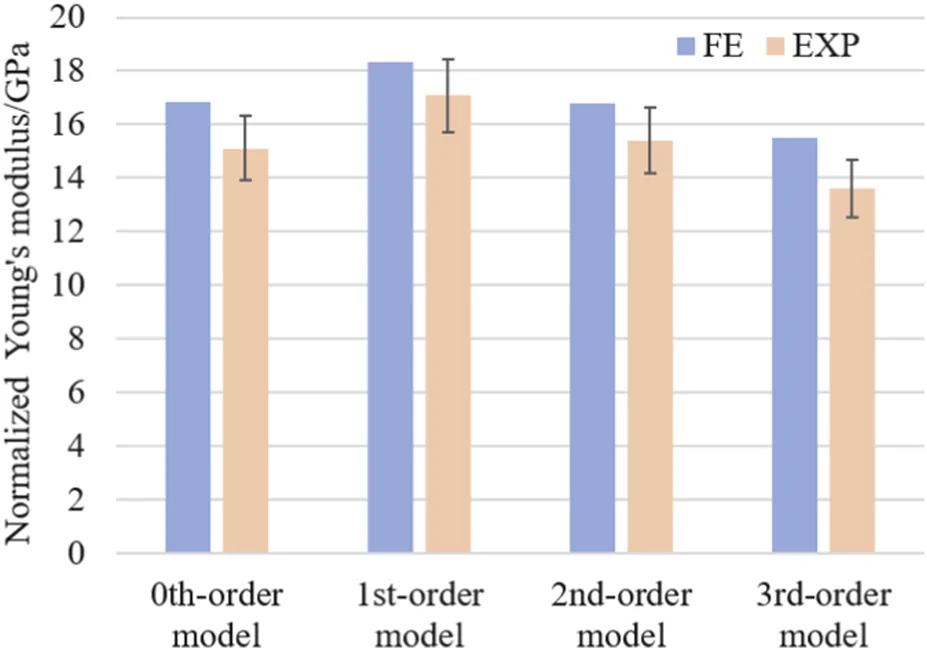
Young’s modulus derived from uniaxial compression tests and finite element simulations of the four biomimetic scaffolds.

This study demonstrates that optimizing fractal parameters enables the achievement of an optimal mechanical matching between Young’s modulus and compressive strength, thereby making the mechanical properties of bone implants more closely resemble those of native bone. In clinical practice, based on the specific characteristics of individual patients with bone defects, porous bone scaffolds can be effectively tailored to meet patients’ personalized requirements by optimizing fractal parameters. This underscores the distinct advantages of tree-like fractal-structured porous bone scaffolds.

## 4 Conclusion

This study proposes a design paradigm for biomimetic porous bone scaffolds with a tree-like fractal structure. A comprehensive workflow including biomimetic design and 3D printing fabrication of porous scaffolds is established. Through theoretical analysis, 3D printing fabrication, and mechanical experiments, the porosity characteristics and mechanical behaviors of the tree-like fractal biomimetic scaffolds were systematically investigated, and the research conclusions are as follows.

1. A systematic theoretical design framework for fractal scaffolds was established, which offers immense potential for the development of biomimetic bone implants. The spatial distribution and morphological configuration of bone trabecular are significantly governed by fractal variables. By adjusting the geometric dimensions and fractal parameters of bone trabeculae based on the structural morphology of patient bone defects, personalized porous biomimetic bone scaffolds can be effectively tailored.
2. Optimization of fractal parameters enables radial gradient control of porosity in porous biomimetic scaffolds. Fractal order *n* serves as a critical determinant of the scaffold’s porosity and radial gradient. Scaffolds designed with second and third fractal orders possess highly porous internal architectures, with a structure analogous to that of natural trabecular bone tissues. In contrast, the peripheral regions of the scaffolds present compact and low-porosity microstructures. Such morphological characteristics replicate the structural configuration of native cortical bone, thereby offering stable mechanical support for tissue reconstruction and regeneration.
3. Tree-like fractal porous bionic scaffolds exhibit excellent manufacturability. The fabricated scaffolds demonstrate a continuous radial gradient porosity distribution, with clearly distinguishable pores in their vicinity that show no blockage or collapse. This structural integrity lays a solid foundation for the further clinical popularization and application of biomimetic fractal scaffolds in bone repair engineering.
4. The layered architecture of tree-like biomimetic scaffolds enables enhanced resistance to axial loads. The fracture path of tree-like fractal biomimetic scaffolds forms an angle of approximately 45° relative to the scaffold axis, which can effectively inhibit the axial propagation of cracks. Furthermore, the layered structure of these biomimetic porous scaffolds provides an expanded contact interface and more uniform stress distribution at the scaffold–native bone interface.
5. Biomimetic fractal scaffolds exhibit the typical mechanical advantage of high strength paired with low Young’s modulus. Experimental results indicate that, among the fractal porous scaffolds, the third-order structure possesses superior compressive strength and exhibits a significantly reduced elastic modulus relative to other fractal structures.

## Data Availability

The original contributions presented in the study are included in the article/supplementary material, further inquiries can be directed to the corresponding author.
